# Nosocomial sinusitis in an Intensive Care Unit: a microbiological study

**DOI:** 10.1590/S1808-86942011000100017

**Published:** 2015-10-19

**Authors:** Leonardo Lopes Balsalobre Filho, Fernando Mirage Jardim Vieira, Renato Stefanini, Ricardo Cavalcante, Rodrigo de Paula Santos, Luis Carlos Gregório

**Affiliations:** 1 Master's degree student, Otorhinolaryngology and Head & Neck Surgery Department, São Paulo Federal University (UNIFESP); 2 Otorhinolaryngology and Head & Neck Surgery Department, São Paulo Federal University (UNIFESP); 3 Otorhinolaryngology and Head & Neck Surgery Department, São Paulo Federal University (UNIFESP); 4 Medical resident in Otorhinolaryngology, UNIFESP; 5 Otorhinolaryngology and Head & Neck Surgery Department, São Paulo Federal University (UNIFESP). Head of the Rhinology Unit, Otorhinolaryngology Department, UNIFESP; 6 Otorhinolaryngology and Head & Neck Surgery Department, São Paulo Federal University (UNIFESP). Head of the Rhinolaryngology Discipline Otorhinolaryngology Department, UNIFESP

**Keywords:** microbiology, intensive care units, sinusitis

## Abstract

Nosocomial sinusitis is a common complication of patients in ICUs. Its diagnosis is important, and early treatment is required to avoid serious complications such as pneumonia, sepsis, meningitis, and intracranial abscesses.

**Aim:** To identify the germs causing sinusitis in ICUs by nasal swabs and maxillary sinus puncture, and to correlate these results.

**Methods:** ICU patients with a diagnosis (CT confirmed) of maxillary sinusitis underwent nasal swab and puncture of the sinus to collect material for culture and antibiogram.

**Results:** This study evaluated 22 patients. The microbial agent isolated in the swab correlated with the agent in the puncture in 14 of 22 cases (63%). Gram-negative bacteria were the most frequent, as follows: Pseudomonas aeruginosa (29% of punctures), following by Proteus mirabillis (26%) and Acinetobacter baumanni (14%). The resistance index in the antibiogram was high to antibiotics.

**Conclusion:** Maxillary sinus puncture of ICU patients with sinusitis appears to be the best method for identifying bacteria; antibiograms demonstrate resistance to therapy. The swab has little diagnostic value; the correlation was 63%. It may be used when sinus puncture is contraindicated.

## INTRODUCTION

Rhinosinusitis is a frequent complication in intubated patients.^1^, ^2^, ^3^ Ventilation-associated pneumonia in longterm intubated intensive care patients may be avoided by systematically searching for and treating nosocomial rhinosinusitis.^4^ Ventilation-associated pneumonia increases significantly the hospital stay and intensive care unit (ICU) mortality.^5^

Nosocomial rhinosinusitis in ICU is often underestimated and undiagnosed.^6^ It is difficult to diagnose in ICUs; the diagnosis is based on computed tomography (CT) radiologic signs and on isolating the offending microorganism from cultures of pus collected by transnasal puncture of facial sinuses.^1,^^4,^^7,^^8^ Transnasal puncture is important for diagnosing and treating rhinosinusitis.^8^ CT is the method of choice for assessing the nasosinusal cavity.^9^

Antral puncture is a useful tool for monitoring these patients - it is both diagnostic and therapeutic. An advantage of puncture is that it does not require the patient to be in a surgical theater; it may be done on the bedside with local anesthesia.^10,^^11^ Rouby et al. reported successful control of fever in 67% of cases by removing infected material from the maxillary sinus followed by lavage with saline solution.^1^ A limitation of this procedure is that it is applicable only to the maxillary sinuses; often there is posterior rhinosinusitis.^1,^^12^

The microbiological agents found in nosocomial rhinosinusitis in ICU patients vary. Multibacterial infections are the most common - there may be two or three concomitant species. Most studies have shown that Gram positive organisms are prevalent (*Staphylococcus aureus, Streptococcus pneumoniae, Enterococcus faecalis*); Gram negative bacteria may also be found (*Pseudomonas aeruginosa, Acinetobacter baumanni, Proteus mirabilis* and others).^13^, ^14^, ^15^, ^16^, ^17^, ^18^ Le Moal showed a high incidence of anaerobic organisms, which may be found in up to 60% of cases (*Prevotella sp, Fusobacterium nucleatum* and *Peptostreptococcus anaerobius*).^19^

The treatment of rhinosinusitis in ICUs should be started promptly because it is often associated with mechanical ventilation-associated pneumonia; this condition may lead to sepsis and death.^19^, ^20^, ^21^ At present, treatment starts with removal of foreign bodies in the nose (tubes and catheters), nasal vasoconstrictors, and antibiotics. Therapeutic failures requires antral puncture of the maxillary sinuses followed by lavage with a saline solution. If failure persists, sinusectomy in the surgical theater to drain all affected sinuses may be indicated.^1,^^13,^^22^ Humphrey has reported that fever regresses within four days after antral puncture, and within 36 hours after surgery.^23^

## OBJECTIVE

The purpose of this study was to identify the causative agents of infectious rhinosinusitis in ICUs by using nasal swabs and maxillary sinus puncture, and to correlate these results among each other and with the antibiogram.

## MÉTODO

A cross-sectional contemporary cohort study was made of patients seen at ICUs of a hospital, in which a diagnosis of infectious sinus disease was made.

The institutional review board approved this study (no. 1208/07). A legal representative of each patient enrolled in this study signed a free informed consent form.

The inclusion criteria aimed to enroll patients with evidence of infectious sinus disease acquired at an ICU, and were as follows:

• ICU stay over 48 hours;
• fever starting after 24 hours of admittance into the ICU;
• a clinical/radiologic diagnosis of infectious sinus disease;
• absence of other infectious sites;
• other infection sites controlled and discarded by intensivists.

Patients that did not met these criteria were excluded from this study, as were patients with a diagnosis of acute or chronic rhinosinusitis or fever before admittance, which did not fall into the focus on nosocomial disease in this study.

The ICU had a protocol for investigating fever, consisting of a blood culture, urine culture, tracheal aspirate culture, a chest X-ray, CSF culture, and CT of the paranasal sinuses.

Paranasal sinus CT (axial and reconstruction of coronal sections) was used as the radiologic parameter for diagnosis. Maxillary sinus opacification (uni- or bilateral) and a air-fluid level, or thickening of the mucosa (≥ 6 mm) was considered as suggesting infection.

Culture and antibiogram was done of middle turbinate secretion collected with a sterile swab guided endoscopically towards the ostiomeatal complex of the affected maxillary sinus.

Material from within the maxillary sinus was collected by puncture; the patient was placed in dorsal decubitus in the ICU bed. Antisepsis was made of the facial area. Patients were sedated with midazolam and fentanyl at individualized doses. Lower airway protection was verified by checking the orotracheal or tracheostomy tube balloon.

Maxillary sinus puncture was done from the lower meatus, using a curved trochar, following infiltration of the caudal portion of the lower turbinate with xylocaine and 1:100.000 vasoconstrictor, and cottonoids with adrenalin placed in the lower meatus. After collecting secretions, the sinuses were irrigated with 0.9% saline solution.

The puncture material was sent for microbiological studies and identification of aerobic microorganisms and fungi, and for the antibiogram.

## RESULTS

This study included 22 patients diagnosed with maxillary nosocomial rhinosinusitis, in whom the procedures were carried out.

All samples sent for microbiologic studies had bacterial growth. On swabs, more than one type of bacteria was present in 8 of 22 cases (37%); the corresponding number in punctures was 9 of 22 cases (41%).

There was exact agreement in swab and puncture bacteria in 14 of 22 cases (63%).

**Figure 1:**
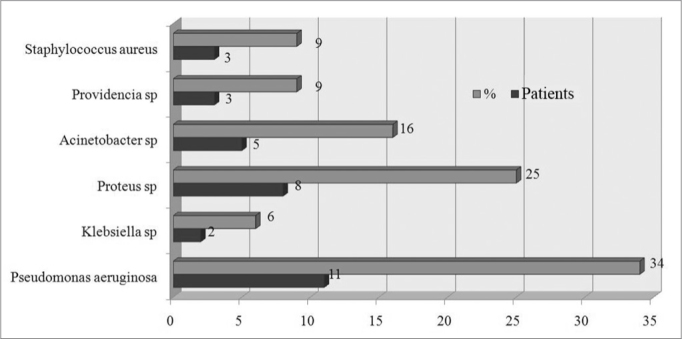
Bacteria species isolated from the culture of a secretion obtained from a middle meatus swab.

**Figure 2:**
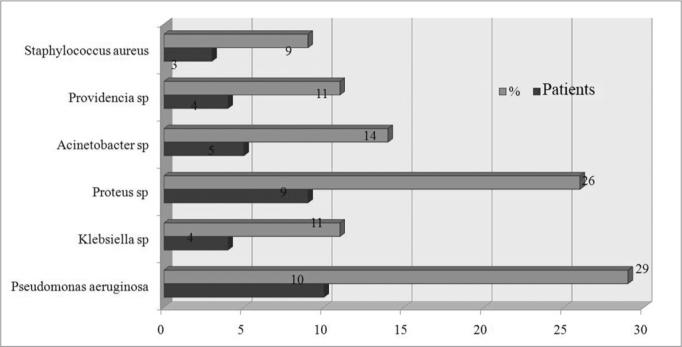
Bacteria species isolated from the culture of a secretion obtained through antral puncture.

Figures 1 and 2, and table 1 present the bacterial species in punctures, swabs, and the resistance to antibiotics, respectively.

**Table 1:** Antibiotic drug resistance tested in cultures of material collected by antral puncture

Antibiotic	%	Antibiotic	%
Cefalotin	100	Ciprofloxacin	42,8
Ceftriaxone	57,1	Levofloxacin	57,1
Ceftazidime	57,1	Meropenem	42,8
Cefepime	71,4	Imipenem	42,8
Gentamicin	42,8	Vancomicin	42,8

## DISCUSSION

Nosocomial rhinosinusitis was first described by Arens et al^24^ in 1974. The incidence of rhinosinusitis in ICUs ranges from 0 to 100%, depending on the patient population and the definition of rhinosinusitis that is used.^25^, ^26^, ^27^

Infectious sinus disease is strongly associated with pneumonia; there is microbiological correlation between both conditions, and the risk of lung infection is nearly fourfold in patients with rhinosinusitis, especially when caused by *Pseudomonas aeruginosa*, *Acinetobacter sp* or *Staphylococcus aureus*.

The microbiology of these infectious differs from that of common sinus infections; published studies have suggested that Gram negative bacteria, anaerobes and fungi are more common. Infection by anaerobic bacteria is unclear, with conflicting results, in the literature. In the present study, Gram negative bacteria predominated as causative agents of infectious rhinosinusitis. Absence of anaerobes in this study may be explained by the fact that the laboratory in which microbiological testing was done did not have the required techniques for investigating anaerobic microorganisms, because of the difficulty in carrying out this type of testing. Conflicting results in the literature may possibly be due to this same technical issue for culturing anaerobes. Nevertheless, the presence of anaerobic bacteria in this disease should not be underestimated.

Many patients in ICUs are started empirically on antibiotics on the first day of fever regardless of whether an infection site has been located or not. The results of blood or urine cultures, which should be done and not underestimated, may guide or require changes in antimicrobial therapy.

Kountakis Skoulas et al. (2002) found little association between the bacteria found in middle meatus lavage and those causing sinus disease (collected by sinus puncture) in the same patient.^17^ We found a positive association in 63% of cases, which concurs with Casiano et al. (2001) - 53% agreement in the results of microbiological studies comparing cultures of material collected by antral puncture and mucosal fragments from the ostiomeatal complex.^28^

We believe that the nasal swab is not the best method for diagnosing pathogens in nosocomial rhinosinusitis in ICUs; the reasonable correlation we found in the present study suggests that this method may be reserved for patients whose clinical status precludes invasive procedures - such as patients with blood dyscrasia. In spite of aseptic procedures, coated swabs, and careful handling to avoid touching other structures in the nasal fossa, this method is much more prone to contamination.

Microbiological study of material collected by direct puncture of the sinus is the best method to guide antibiotic therapy. Pus from the sinus is the study material, thereby reducing the possibility of contamination, which could yield false positive results.

We found a high antibiotic resistance rate, at times reaching 100%. Thus, the antibiogram of sinus material is extremely important to help decide which antibiotic should be used. Empiric antibiotic therapy often means that ineffective drugs will be used, which maintains infection and may result in higher rates of pneumonia, sepsis, meningitis, and intracranial abscesses, thereby increasing the morbidity and mortality of these patients.

## CONCLUSION

We concluded that direct puncture of the maxillary sinus in patients with nosocomial rhinosinusitis is the best method to identify disease causing bacteria, and that an antibiogram of this material may help guide antibiotic therapy.

The swab was shown to have little diagnostic value. The agreement rate was 63%, suggesting that it may be reserved for patients where sinus puncture is contraindicated.

Gram negative bacteria were the most frequent infectious agents in the following order: *Pseudomonas aeruginosa* (29% of punctures), *Proteus mirabillis* (26%) and *Acinetobacter baumanni* (14%).

The bacterial resistance rate was high in the antibiogram, in some cases reaching 100% (Table 1).
